# The likelihood of total knee arthroplasty following arthroscopic surgery for osteoarthritis: a systematic review

**DOI:** 10.1186/s12891-017-1765-0

**Published:** 2017-10-04

**Authors:** Amelia R. Winter, Jamie E. Collins, Jeffrey N. Katz

**Affiliations:** 1 Orthopaedic and Arthritis Center for Outcomes Research (OrACORe), Department of Orthopedic Surgery, Boston, MA USA; 2 Division of Rheumatology, Immunology and Allergy, Brigham and Women’s Hospital, 60 Fenwood St, Suite 5016, Boston, MA 02115 USA; 3 Harvard Medical School, Boston, MA USA; 4 Departments of Epidemiology and Environmental Health, Harvard T. H. Chan School of Public Health, Boston, MA USA

**Keywords:** Osteoarthritis, Total knee arthroplasty, Arthroscopic partial meniscectomy, Arthroscopy

## Abstract

**Background:**

Arthroscopic surgery is a common treatment for knee osteoarthritis (OA), particularly for symptomatic meniscal tear. Many patients with knee OA who have arthroscopies go on to have total knee arthroplasty (TKA). Several individual studies have investigated the interval between knee arthroscopy and TKA. Our objective was to summarize published literature on the risk of TKA following knee arthroscopy, the duration between arthroscopy and TKA, and risk factors for TKA following knee arthroscopy.

**Methods:**

We searched PubMed, Embase, and Web of Science for English language manuscripts reporting TKA following arthroscopy for knee OA. We identified 511 manuscripts, of which 20 met the inclusion criteria and were used for analysis. We compared the cumulative incidence of TKA following arthroscopy in each study arm, stratifying by type of data source (registry vs. clinical), and whether the study was limited to older patients (≥ 50) or those with more severe radiographic OA. We estimated cumulative incidence of TKA following arthroscopy by dividing the number of TKAs among persons who underwent arthroscopy by the number of persons who underwent arthroscopy. Annual incidence was calculated by dividing cumulative incidence by the mean years of follow-up.

**Results:**

Overall, the annual incidence of TKA after arthroscopic surgery for OA was 2.62% (95% CI 1.73–3.51%). We calculated the annual incidence of TKA following arthroscopy in four separate groups defined by data source (registry vs. clinical cohort) and whether the sample was selected for disease progression (either age or OA severity). In unselected registry studies the annual TKA incidence was 1.99% (95% CI 1.03–2.96%), compared to 3.89% (95% CI 0.69–7.09%) in registry studies of older patients. In unselected clinical cohorts the annual incidence was 2.02% (95% CI 0.67–3.36%), while in clinical cohorts with more severe OA the annual incidence was 4.13% (95% CI 1.81–6.44%). The mean and median duration between arthroscopy and TKA (years) were 3.4 and 2.0 years.

**Conclusions:**

Clinicians and patients considering knee arthroscopy should discuss the likelihood of subsequent TKA as they weigh risks and benefits of surgery. Patients who are older or have more severe OA are at particularly high risk of TKA.

**Electronic supplementary material:**

The online version of this article (10.1186/s12891-017-1765-0) contains supplementary material, which is available to authorized users.

## Background

Osteoarthritis (OA) is a debilitating disease, affecting over 40 million people in the United States [1, 2]. Of those affected, approximately 14 million have symptomatic knee OA [3], which presents with pain, loss of knee joint function, and loss of valued activities. In addition, about 90% of those with symptomatic knee OA have meniscal tears (MT) documented on magnetic resonance imaging (MRI) [4]. However, no available treatments modify the structural progression associated with OA. Symptoms are generally managed with conservative therapies (e.g., nonsteroidal anti-inflammatory drugs (NSAIDs), exercise, physical therapy). Patients and their physicians often turn to surgical treatments to address progressive pain and disability, including arthroscopy and total knee arthroplasty (TKA).

Over 600,000 arthroscopic partial meniscectomies (APM) are performed each year in the United States [5], most commonly on persons over 45 with MT [5, 6]. The benefit of arthroscopic surgery in patients with OA is uncertain and debated. Moseley et al. showed that sham surgery and arthroscopic surgery for OA had similar pain relief and functional improvement up to 2 years post-surgery [7]. Kirkley and colleagues showed that arthroscopy and a conservative exercise regimen had similar symptomatic and functional outcomes in persons with knee OA [8]. With respect to MT in the setting of OA, several trials demonstrated that surgery was not superior to nonoperative therapy or sham surgery in intention to treat analyses [9–12], while one trial showed a benefit for surgery [13]. Thus, arthroscopic surgery is felt to be ineffective for OA per se, while the effectiveness of APM in persons with MT and concomitant OA is debated [14].

Often, people who undergo arthroscopic surgery for osteoarthritis progress to TKA. While some studies suggest that up to 20% of patients undergo TKA within one year of arthroscopy [15], other studies have shown TKA rates under 5% [16]. The various studies of the rate of TKA after arthroscopy have not been summarized, to our knowledge. Such a summary of the risk of TKA following arthroscopy and the duration between arthroscopy and TKA would be helpful for clinicians to better advise patients and their families on appropriate treatments plans. Surgery is expensive – about $2 billion dollars are spent on arthroscopy for OA [17] and over $10 billion dollars are spent on TKAs each year [18]. Therefore, improved knowledge of the risk for TKA following arthroscopy could also lead to better resource allocation for OA.

We performed a systematic review of the literature on the risk of TKA following arthroscopic surgeries for OA. We expected to see older patients and those with more severe OA, progress to TKA more quickly after surgery.

## Methods

### Definition of search terms

A search by title was performed on PubMed, Embase, and Web of Science using the major search terms: *osteoarthritis, knee; arthroscopy; and arthroplasty* (see Additional file 1: Table S1 for search strings). The search was performed in September 2016 and titles were downloaded to EndNote. One reviewer (ARW) manually screened titles for inclusion and exclusion criteria, arriving at a final list of titles. For these titles, the reviewer assessed abstracts for inclusion and exclusion criteria. For each abstract that was not excluded, the full manuscript was read to determine ultimate inclusion in the final analysis. A second reviewer confirmed that the final selected manuscripts met inclusion criteria.

### Inclusion and exclusion criteria

We sought studies investigating the rate of arthroplasty after arthroscopic knee surgery for osteoarthritis. Therefore, inclusion criteria included: English language and human studies on the risk of TKA occurring following arthroscopic procedures for knee OA. Manuscripts were excluded if they were duplicates, written in a language other than English, or conducted on animals. Studies were also excluded if they examined only arthroscopy or arthroplasty rather than the risk of arthroplasty following arthroscopy. Case studies and studies with cohorts of mean age < 40 were also excluded. Confusion regarding inclusion of a study was resolved by consulting with the senior author (JNK).

### Data abstraction

From the manuscripts, the reviewer (ARW) extracted the following information (if available): author, year, title, administrative data (e.g., clinical cohort or registry), country, patient selection criteria (e.g. age, KL grade), subgroup information, size of analysis group, mean age, analysis method (e.g., cumulative incidence), duration between arthroscopy and TKA, duration of follow-up, percentage of TKA, and study arm population description. Another reviewer abstracted key data (e.g., country, administrative data, patient selection criteria, follow-up years, analysis group, and total TKA numbers) from the included studies. The results from both abstractions were compared and found to be the same.

### Categorization of studies

We examined the cumulative incidence of TKA following knee arthroscopy in specific patient subgroups. These included source of data (administrative data registries vs. clinical cohort studies), OA severity, older age (e.g., selection for population ≥ 50), and country. Some of the clinical cohort studies recruited patients with advanced OA, (i.e., KL grade ≥ 3 or Outerbridge score ≥ 2). We defined these study arms as “Clinical Cohort – More Severe OA.” One study (Lyu et al., 2015) was included among the “Clinical Cohort – More Severe OA” group as its patient population was over 75% KL grade 3 or higher. Some registry studies were restricted to subjects with age greater than 50. We referred to these as “Registry – Older Age.” We created a final categorization combining the source of data and selection criteria: “Registry – Unselected,” “Registry – Older Age,” “Clinical Cohort – Unselected,” and “Clinical Cohort – More Severe OA.” For countries, we combined England and Scotland as “U.K.”

### Quality assessment

We used the Quality Assessment Tool for Observational Cohort and Cross-Sectional Studies developed by investigators at the National Heartt, Lung and Blood Intitute (NHLBI), based upon work done at the Agency for Health Care Research and Quality. (https://www.nhlbi.nih.gov/health-pro/guidelines/in-develop/cardiovascular-risk-reduction/tools/cohort) [19]. This measure includes 14 items relevant to the quality of cohort studies, with emphasis on explicit specification of sample characteristics, exposures, primary outcomes and potential confounders. Two authors performed the assessment independently and resolved any disagreements.

### Analyses

For studies that did not provide cumulative incidence data, we calculated cumulative incidence using the number of TKAs divided by the number of arthroscopic patients included for follow-up analysis. We first examined the association between the type of data source – registry vs. cohort – and TKA rates. Then, we evaluated the association between study category (“Registry – Unselected,” “Registry – Older Age,” “Clinical Cohort – Unselected,” and “Clinical Cohort – More Severe OA,” as described above) and annual incidence. In secondary analyses, we evaluated the difference between TKA annual incidence in unselected study arms (clinical and registry) vs. selected study arms, regardless of registry status. We compared studies in which mean age of the study was >65 to those with mean age < 65.

We divided cumulative incidence of TKA by mean years of follow-up to obtain an annual incidence estimate. We computed exact confidence intervals for each yearly incidence value. We used a logistic random-effects model to create an overall combined estimate of annual TKA incidence across all studies and to evaluate the effect of study-level characteristics on TKA incidence. This approach allows for studies with zero cells (i.e., 0% incidence rate) without requiring an ad-hoc adjustment [20]. All analyses were conducted using SAS 9.4 (SAS Institute, Cary NC).

## Results

Five hundred eleven unique articles were found using our search terms and three search engines. After screening the titles, we were left with 328 articles whose abstracts were subsequently reviewed. Over half of the articles excluded did not have arthroscopic surgery before TKA (36%) or did not report TKA (24%). Fifty-five articles underwent full article review. Thirty-five of the manuscripts were excluded from our analysis: 11 were not written in English, 2 did not report on arthroscopic procedures before TKA, 3 did not report on TKA, and 19 were excluded for other reasons, such as mixed cohort (e.g., OA and post-traumatic arthritis), secondary sources, or insufficient data on methodology.

**Fig. 1:**
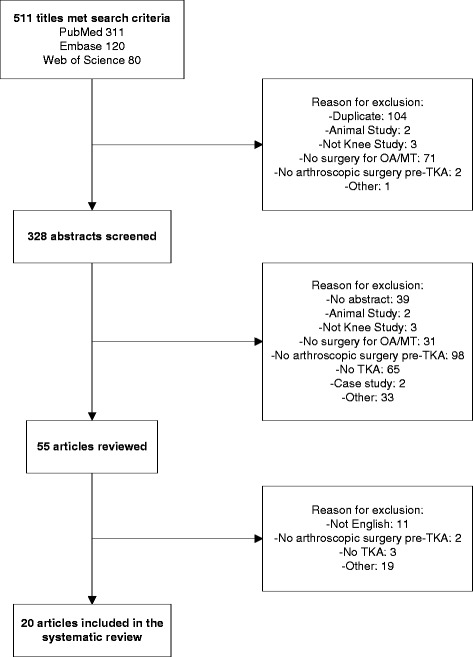
Search and selection process

These exclusions left 20 articles for the analysis (Fig. 1). The 20 studies contained 28 unique study arms (Table 1). The 28 study arms were reported from eight countries. The U.S.A. accounted for 15 of the 28, the U.K. for 5, Canada for 3, and Australia, Belgium, Italy, South Korea, and Taiwan for one each. The quality assessment documented relatively little variability in quality. Essentially all the studies stated the research question clearly, specified the population and defined the exposure and outcome explicitly. Only one study provided a power calculation. Rates of participation among eligible subject and rates of follow up were generally high, particularly for administrative data studies in which participation and follow-up rates are typically 100%. Some of the quality items did not apply to the studies we reviewed because all subjects in our studies were ‘exposed’ (had arthroscopy).

**Table 1:** Characteristics of included studies

			Author and Year	Country	Follow-Up (Years)	Mean Duration (years)	Analysis Group	Total TKA	Annual Incidence (%)	Lower 95% CI	Upper 95% CI
Clinical Cohort	Selected for More Severe OA	KL ≥ 3	Bernard et al. (2004) [22]	U.K.	5	Unknown	100	11	2.20%	1.10%	3.90%
		KL = 4	Bin et al. (2008) [34]	South Korea	4	4	68	4	1.36%	0.37%	3.43%
		Outerbridge ≥2	Koyonos et al. (2009)^a^ [16]	U.S.A.	1	0.5	30	1	3.33%	0.08%	17.22%
						0	29	0	0.00%	0.00%	11.94%
		KL ≥ 3	Lyu et al. (2015) [35]	Taiwan	1	1	844	116	13.74%	11.49%	16.25%
		KL = 4	Pearse and Craig (2003) [36]	U.K.	4	4	126	39	7.14%	5.13%	9.64%
		KL ≥ 3	Rand et al. (1985) [37]	U.S.A.	2	0.5	87	2	1.15%	0.14%	4.09%
		Outerbridge ≥2	Skedros et al. (2014) [30]	U.S.A.	3	3	42	11	8.73%	4.44%	15.08%
		KL ≥ 3	Steadman et al. (2013) [31]	U.S.A.	10	4.4	69	43	6.23%	4.55%	8.30%
	Unselected					2	8	0	0.00%	0.00%	8.81%
			Jackson et al. (2003)^a^ [25]	U.S.A.	5		32	0	0.00%	0.00%	2.28%
							39	3	1.54%	0.32%	4.43%
							42	12	5.71%	2.99%	9.77%
			McGinley et al. (1999) [27]	U.S.A.	13	7	91	30	2.50%	1.69%	3.55%
			Raaijmaakers et al. (2010) [28]	Belgium	3	1	183	40	6.83%	4.92%	9.18%
			Sansone et al. (2015) [29]	Italy	20	13.3	75	12	0.80%	0.41%	1.39%
Registry	Selected for Older Age	Age > 60	Dearing et al. (2010) [38]	U.K.	9	6	3033	800	2.93%	2.73%	3.14%
		Age > 65	Johanson et al. (2011) [26]	U.S.A.	10	9	40,804	13,261	3.25%	3.20%	3.30%
		Age > 50	Wai et al. (2002) [32]	Canada	3	3	6212	1146	6.15%	5.81%	6.50%
	Unselected		Adelani et al. (2016)^a^ [39]	U.S.A.	4	2	6972	266	0.95%	0.84%	1.07%
							10,645	496	1.16%	1.06%	1.27%
			Fedorka et al. (2014) [23]	U.S.A.	5	Unknown	159,975	8319	1.04%	1.02%	1.06%
			Harris et al. (2013) [40]	Australia	8	2	121,115	9110	0.94%	0.92%	0.96%
				U.K 1993		Unknown	6158	985	3.20%	3.00%	3.40%
							9048	1728	3.82%	3.64%	4.00%
			Hawker et al. (2008)^a^ [24]	UK 1997 Canada 1993	5						
							3803	745	3.92%	3.65%	4.20%
				Canada 1997							
							3425	712	4.16%	3.86%	4.47%
			Zikria et al. (2016) [41]	U.S.A.	7	Unknown	842	131	2.22%	1.86%	2.63%

^a^Studies contain multiple unique study arms, which were separated for our analysis; Jackson rows: Severity stages I, II, III, IV

**Fig. 2:**
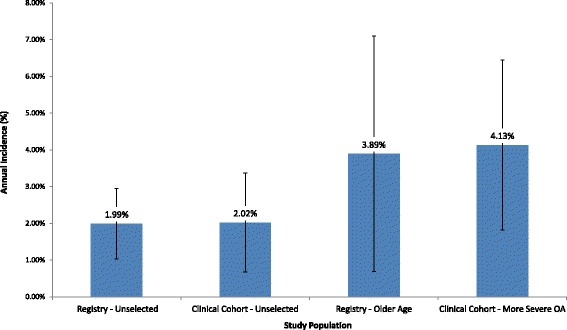
Mean Annual Incidence of Registry – Unselected, Clinical Cohort – Unselected, Registry – Older Age, and Clinical Cohort – More Severe OA. Each bar represents the estimated yearly incidence of TKA from the logisitc random effects model. The vertical lines represent the 95% confidence intervals

Overall, the yearly incidence for TKA after arthroscopic surgery for OA was 2.62% (95% CI 1.73–3.51%). The mean and median duration between arthroscopy and TKA (years) were 3.4 and 2.0 years. From our 28 study arms, we identified sixteen clinical cohorts and twelve registry samples. The clinical cohort studies had a yearly TKA incidence of 2.94% (95% CI 1.54–4.33%), compared to the registry studies, which had an incidence of 2.36% (95% CI 1.26–3.46%) (*p* = 0.5048). We examined separately the risk of TKA in four distinct subgroups: “Registry – Unselected,” “Registry – Older Age,” “Clinical Cohort – Unselected,” and “Clinical Cohort – More Severe OA.” The four subgroups are shown in Fig. 2.

### Registry - unselected

A total of nine study arms were unselected registries, with a median of 6972 (range 842–159,975) patients per study arm (Table 1). Of these, the average yearly incidence for TKA was 1.99% (95% CI 1.03–2.96%) (Fig. 2).

### Registry – Older age

A total of three study arms were registries using data from patients ≥50 years old, with a median of 6212 (range 3033–40,804) patients per study arm (Table 1). Of these studies, the average yearly incidence for TKA was 3.89% (95% CI 0.69–7.09%) (Fig. 2).

### Clinical cohort – Unselected

A total of seven study arms were unselected clinical cohorts, with a median of 42 (range 8–183) patients per study arm (Table 1). Of these studies, the average yearly incidence for TKA was 2.02% (95% CI 0.67–3.36%) (Fig. 2).

### Clinical cohort – More severe OA

A total of nine study arms were clinical cohorts selecting for patients with more severe OA on the basis of KL grade or Outerbridge score, with a median of 69 (range 68–844) patients per study arm (Table 1). Of these studies, the average yearly incidence for TKA was 4.13% (95% CI 1.81–6.44%) (Fig. 2).

### Comparisons: Age and OA severity

We evaluated the association between TKA incidence and study inclusion criteria using a logistic random-effects model. We found that selected studies - those that selected subjects based on OA severity or age - were twice as likely to undergo TKA compared to unselected studies (4.05% compared to 2.00%; *p* = 0.0243). Studies of subject with a mean age of less than 65 had a yearly incidence of 1.87% (95% CI 1.16–2.57%) compared to 5.13% (95% CI 2.61–7.64%) for those with mean age over 65. This difference was statistically significant (*p* = 0.0027).

## Discussion

We evaluated published literature on the risk of TKA in patients undergoing knee arthroscopy. A concern about the use of arthroscopic surgery in the setting of OA and OA with meniscal tear is that APM may lead to more rapid OA progression, leading to TKA more quickly [1, 15]. We found that on average the risk of TKA following arthroscopy was about 2% per year and that the mean and medican duration between arthroscopy and TKA were 3.4 and 2.0 years respectively. Further, study arms of patients who were older or had more advanced radiographic OA at the time of arthroscopy had two-fold higher risk of TKA than unselected study arms. These findings should be viewed in the context of other documented risk factors for OA progression including older age, female gender, varus and valgus malalignment and bone marrow lesions, among others [21].

Our findings are consistent with studies showing that OA severity and age are associated with TKA [22–32]. Indeed, surgeons may be reluctant to offer TKA to younger patients, because they face a risk of a revision TKA. Advanced OA is a typical indication for TKA, as embodied in guidelines such as those of the American Academy of Orthopaedic Surgeons [33].

Our study must be interpreted in the context of several limitations. The clinical cohort data provided insight into the KL grades and Outerbridge scores of patients whereas registry data included age but no information on OA severity nor on the details of surgery. The component studies did not perform analyses of subgroups that might be prognostically distinct, such as athletes and non-athletes, or males and females. Similarly, over half of the countries contributed just one cohort. This precludes meaningful analysis of between-country differences. While we performed replicate abstractions of all papers we did not repeat the screening of titles and abstracts in duplicate, creating the theoretical risk of our missing an eligible paper. As reflected in our quality assessment, the studies consistently defined the exposure and outcome explicitly. Since most of the larger studies used administrative data, the follow-up rates were generally 100%. We note as well that some patients with a medical ‘need’ for TKA (symptomatic, advanced OA) may not have received the procedure because of their own preferences or the practice styles of their physicians or still other reasons. When TKA is used as a health outcome, the role of these patient, physician and health system factors may attenuate the risk associated with specific variables such as prior arthroscopic surgery.

To the best of our knowledge, this is the first systematic review to analyze the yearly TKA incidence rate for those having undergone arthroscopic surgery for knee OA. Quality assessment of the studies generally reflected consistent specification of exposures, outcomes, and study samples and high rates of participation and follow-up. The findings suggest that OA patients undergoing arthroscopy and their physicians should anticipate an annual rate of TKA on the order of 2%, with higher rates among older patients and those with more advanced OA. These findings should be shared with patients when clinicians discuss the advantages and drawbacks of arthroscopy.

## Conclusion

Clinicians and patients considering knee arthroscopy should discuss the likelihood of subsequent TKA as they weigh risks and benefits of surgery. Patients who are older or have more severe OA are at particularly high risk of TKA.

## Additional file

Additional file 1: Table S1. Search Strings by Search Engines. Table of bibliographic search terms used to identify candidate articles. (DOCX 13 kb)

## Abbreviations

KL: Kellgren-Lawrence
MRI: Magnetic resonance imaging
MT: Meniscal tear
NSAIDs: Nonsteroidal anti-inflammatory drugs
OA: Osteoarthritis
tka: Total knee arthroplasty
