# Correction to: The likelihood of total knee arthroplasty following arthroscopic surgery for osteoarthritis: a systematic review

**DOI:** 10.1186/s12891-022-05344-3

**Published:** 2022-07-25

**Authors:** Amelia R. Winter, Jamie E. Collins, Jeffrey N. Katz

**Affiliations:** 1 Orthopaedic and Arthritis Center for Outcomes Research (OrACORe), Department of Orthopedic Surgery, Boston, MA USA; 2 Harvard Medical School, Boston, MA USA; 3 Division of Rheumatology, Immunology and Allergy, Brigham and Women’s Hospital, 60 Fenwood St, Suite 5016, Boston, MA 02115 USA; 4 Departments of Epidemiology and Environmental Health, Harvard T. H. Chan School of Public Health, Boston, MA USA

**Correction to: BMC Musculoskelet Disord 18, 408 (2017)**

**https://doi.org/10.1186/s12891-017-1765-0**

The authors included an article by Lyu [1] in their systematic review [2] and now recognize it should be excluded, as the goal of the Lyu article was not to investigate the annual incidence of total knee arthroplasty (TKA) following traditional arthroscopy.

Several estimates of the annual incidence of TKA following arthroscopy have been recalculated with the data of Lyu [1] excluded. The authors categorized the studies included in the systematic review as registry studies or clinical cohorts. Lyu [1] was categorized as a clinical cohort; thus, the corrections pertain to clinical cohorts and to overall estimates that include both registry studies and clinical cohorts. These recalculations (shown below) correct statements made in the Results section of the original paper and in Figure 2.

The information from Lyu in Table 1 should be modified as follows: Analysis Group (from 844 to 657), Total TKA (116 to 3) and Annual Incidence (from 13.74% to 0.46%).

Following publication of this article [2], the authors report the following Corrections to the main text, where the data of Lyu [1] has been removed from the calculations presented below:

Annual incidence of TKA:

The annual incidence of TKA after arthroscopic surgery for OA has been updated to 2.46% (95% CI 1.68–3.25%).

Annual incidence of TKA by study type (clinical cohort vs. registry):

The clinical cohort studies had a yearly TKA incidence corrected to **2.60% (95% CI 1.38–3.81%)**, compared to the registry studies, which had an updated incidence of 2.36% (95% CI 1.26–3.46%) **(p = 0.7591).**

Annual incidence of TKA by subgroup in clinical cohorts:

In unselected clinical cohorts the annual incidence was updated to 2.02% (95% CI 0.67–3.36%), while in clinical cohorts with more severe OA the annual incidence was corrected to **3.36% (95% CI 1.38–5.34%).**

Comparisons: Age and OA severity

We corrected and found that selected studies – those that selected subjects based on OA severity or age - were more likely to undergo TKA compared to unselected studies **(3.54% compared to 2.00%; p = 0.0626)**
