# Supplementary material for: The likelihood of total knee arthroplasty following arthroscopic surgery for osteoarthritis: a systematic review
Source: BMC Musculoskelet Disord. 2017 Oct 4;18:408. doi: 10.1186/s12891-017-1765-0 (PMC5628417; doi:10.1186/s12891-017-1765-0)
Supplement: Additional file 1: Table S1. — Search Strings by Search Engines. Table of bibliographic search terms used to identify candidate articles. (DOCX 13 kb) [file 12891_2017_1765_MOESM1_ESM.docx]

Table S1. Search Strings by Search Engines

| **PubMed** | **Embase** | **Web of Science** |
| --- | --- | --- |
| ("Osteoarthritis, Knee"[Mesh] OR (osteoarthritis[tiab] AND knee*[tiab])) AND ("Arthroscopy"[Mesh] OR arthroscop*[tiab]) AND ("Arthroplasty, Replacement, Knee"[Mesh] OR (knee*[tiab] AND (arthroplast*[tiab] OR replacement*[tiab]))) | ('knee osteoarthritis'/exp OR (osteoarthritis NEAR/3 knee*):ab,ti) AND ( 'knee arthroscopy'/exp OR arthroscop*:ab,ti) AND ( 'knee arthroplasty'/exp OR (knee* NEAR/3 (arthroplast* OR replacement*)):ab,ti) | TS=("osteoarthritis" NEAR/3 "knee*") AND TS="arthroscop*" AND (TS=("knee*" NEAR/3 "arthroplast*") OR TS=("knee*" NEAR/3 "replacement*")) |
